# Temperature dependence of photosynthetic reaction centre activity in *Rhodospirillum rubrum*

**DOI:** 10.1007/s11120-019-00652-7

**Published:** 2019-07-02

**Authors:** David Kaftan, David Bína, Michal Koblížek

**Affiliations:** 1 Center Algatech, Institute of Microbiology CAS, 37981 Třeboň, Czech Republic; 2 Faculty of Science, University of South Bohemia, 37005 Ceske Budejovice, Czech Republic; 3 Biology Centre, Czech Academy of Sciences, Branišovská 31, Ceske Budejovice, Czech Republic

**Keywords:** Anoxygenic photosynthesis, Electron transfer, Thermostability, Reaction centre, Variable fluorescence

## Abstract

The influence of temperature on photosynthetic reactions was investigated by a combination of time-resolved bacteriochlorophyll fluorescence, steady-state and differential absorption spectroscopy, and polarographic respiration measurements in intact cells of purple non-sulphur bacterium *Rhodospirillum rubrum*. Using variable bacteriochlorophyll fluorescence, it was found that the electron-transport activity increased with the increasing temperature up to 41 °C. The fast and medium components of the fluorescence decay kinetics followed the ideal Arrhenius equation. The calculated activation energy for the fast component was *E*_a1_ = 16 kJ mol^−1^, while that of the medium component was more than double, with *E*_a2_ = 38 kJ mol^−1^. At temperatures between 41 and 59 °C, the electron transport was gradually, irreversibly inhibited. Interestingly, the primary charge separation remained fully competent from 20 to 59 °C as documented by both BChl fluorescence and differential absorption spectroscopy of the P_870_^+^ signal. At temperatures above 60 °C, the primary photochemistry became reversibly inhibited, which was manifested by an increase in minimal fluorescence, *F*_0_, whereas maximal fluorescence, *F*_M_, slowly declined. Finally, above 71 °C, the photosynthetic complexes began to disassemble as seen in the decline of all fluorometric parameters and the disappearance of the LH1 absorption band at 880 nm. The extended optimal temperature of photosynthetic reaction centre in a model species of Rhodospirillales adds on the evidence that the good thermostability of the photosynthetic reaction centres is present across all Alphaproteobacteria.

**Electronic supplementary material:**

The online version of this article (10.1007/s11120-019-00652-7) contains supplementary material, which is available to authorized users.

## Introduction

Phototrophic organisms represent one of the oldest forms of life on Earth. Over the three billion years of evolution (Björn and Govindjee 2009; Nisbet and Sleep 2001), the phototrophs have had to adapt to various environmental conditions including physical (temperature, radiation or pressure) and chemical extremes (desiccation, salinity, pH, oxygen species or redox potential) (Rothschild and Mancinelli 2001). Among them, high temperatures represent one of the main challenges.

In general, temperature has a complex effect on photosynthetic reactions. At lower temperatures (above the freezing point of water), it restricts forward electron transfer, but does not critically affect primary charge separation. On the other hand, higher temperatures can facilitate electron transfer, but if too high, this may alter normal functioning of the photosynthetic reactions, resulting in the disruption and damage of the photosynthetic machinery (Odahara et al. 2011). In our previous study with mesophilic phototrophic Rhodobacterales (class Alphaproteobacteria), we have shown that their reaction centres (RCs) have an optimum of electron transport between 40 and 50 °C (Kaftan et al. 2019). The same optimum was also found for respiration in *Dinoroseobacter shibae* (Kaftan et al. 2019). This was significantly higher than the growth temperature of the respective organisms. Our observation was put in context by the optimal temperature for key enzymes involved in oxidative phosphorylation in mitochondria (Chrétien et al. 2018). The mitochondrial cytochrome *c* oxidases have temperature optima around 50 °C, while ATPase optimum was found around 46 °C (Chrétien et al. 2018). The higher temperature optimum of mitochondrial enzymes was interpreted as a necessary prerequisite for their efficient operation within the highly metabolically active mitochondria that elevate their internal temperature by up to 10 °C above the ambient. It is well established that mitochondria evolved from endosymbiotic Alphaproteobacteria which entered their eukaryotic host. The same electron-transport optimum was also observed in *Erythrobacter* sp. NAP1 (Kaftan et al. 2019). *Erythrobacter* belongs to the order Sphingomonadales within the Alphaproteobacteria, which indicates that the extended temperature optimum of the photosynthetic reactions may be a more general phenomenon.

All these facts suggest that the elevated optima for both respiratory and photosynthetic electron-transport activities are a common trait among all Alphaproteobacteria. To put this hypothesis to the test, we set out to investigate the thermal optimum of photosynthetic reactions in *Rhodospirillum rubrum* strain S1 (Esmarch 1877; Molisch 1907). *R. rubrum* is a common model organism for the investigation of bacterial photosynthesis. It can grow under both photoautotrophic and photoheterotrophic conditions, with a growth optimum of 33 °C (Kaiser and Oelze 1980a; Weaver 1971). Phylogenetically, it belongs to Rhodospirillales that is one of the main orders of Alphaproteobacteria. Also, Rhodospirillales represent the closest branch to Rhodobacterales, the subject of our previous study (Kaftan et al. 2019). The temperature dependence of photosynthetic activity of *R. rubrum* was measured *in vivo* using bacteriochlorophyll (BChl) fluorometry and absorption spectroscopy providing information about primary photochemistry as well as electron-transport activity.

## Materials and methods

### Microbial cultures

*Rhodospirillum rubrum* S1 was purchased from DSMZ culture collection (DSM no. 467). The cells were grown photohetorotrophically under semiaerobic conditions in closed-cap bottles in the organic medium (Cohen-Bazire et al. 1957) at 25 °C with a 12 h light/12 h dark regime. Illumination was provided by a bank of Osram Dulux L 55 W/865 luminescent tubes (spectral temperature of 6500 K) delivering approx. 100 μmol(photon) m^−2^ s^−1^.

### Fluorescence measurements

Infra-red fluorescence measurements were performed using a kinetic fluorometer FL-3000 (Photon Systems Instruments Ltd., Brno, Czech Republic) equipped with the optical unit populated with an array of cyan 505 nm Luxeon Rebel diodes. The infra-red BChl fluorescence signal (λ > 850 nm) was detected using a silicon photodiode registering with a 10 MHz resolution. A thermoregulator TR100 (Photon Systems Instruments Ltd., Brno, Czech Republic) controlled the temperature of the sample in the magnetically stirred cuvette during the experiment. The cells harvested at late exponential phase were diluted with fresh growth medium to 0.2 mg BChl *a* L^−1^ and dark adapted for half an hour at room temperature under aerobic conditions prior to probing their photosynthetic activity. Aliquots of 2.3 mL of the cell suspension were briefly heated/cooled to the chosen temperature and then kept at a constant temperature for 5 min in the dark. The flash fluorescence induction (Nedbal et al. 1999) of BChl fluorescence was elicited by a 140 μs-long square-wave pulse of light with an intensity of ~ 0.1 mol(photon) m^−2^ s^−1^. The minimal BChl fluorescence yield, *F*_0_ registered at the time 1 μs is emitted by the open RCs capable of charge separation. The maximal BChl fluorescence yield, *F*_M_ registered after 140 μs of illumination is emitted by the closed RCs that have undergone charge separation and stabilization of the electron at the quinone primary acceptor Q_A_. Variable fluorescence was calculated as *F*_V_ = (*F*_M_ − *F*_0_), and the yield of primary photochemistry as *F*_V_/*F*_M_. The functional cross-section of the photosynthetic RC and its adjacent light harvesting complexes *σ*_RC_, was deconvoluted from the BChl fluorescence induction kinetics, by numerical fitting of the data to a model of Lavergne and Trissl (1995). The fluorescence relaxation following the maximal BChl fluorescence level *F*_M_ was detected by 800 ns long probing flashlets logarithmically spaced after the saturating pulse (3 flashes per decade, in total 25 data points). The normalized BChl fluorescence decays were fitted with three exponential–decay curves by least square numerical fitting. The rate of fluorescence relaxation was expressed as a sum of three components: *f*(*t*) = *a*_1_ exp(− *k*_1_*t*) +* a*_2_ exp(− *k*_2_ t) +* a*_3_ exp(− *k*_3_*t*) +* F*_0_, where *f*(*t*) is the fluorescence response at time *t*; *k*_1_, *k*_2_, and *k*_3_ represent the rate constants of BChl fluorescence decay components, and *a*_1_, *a*_2_, and *a*_3_ their corresponding amplitudes, respectively. The sum of amplitudes equals the variable fluorescence *F*_V_ = *a*_1_ + *a*_2_ + *a*_3_. To characterize the rate of the RC reopening after the single-turnover saturating flash, we used the parameter RC reopen ing = (*a*_1_*k*_1_ +  *a*_2_*k*_2_ + * a*_3_*k*_3_)/*F*_V_. The individual measurements were conducted over a range from 20 to 80 °C with approx. 3 °C increments with a fresh sample for each individual measurement (i.e. in total 20 measurements). All fluorescence measurements were performed with cells equilibrated with the ambient air, e.g. under fully aerobic conditions.

### Absorption spectroscopy

The steady-state *in vivo* absorption spectra were recorded using a Shimadzu UV 3000 spectrophotometer operating in dual beam mode at 0.5 nm resolution using a 5 nm spectral slit width. The samples were placed in the temperature-controlled cuvette holder heated by a water circulation thermostat. Difference absorption spectroscopy was performed using a modified kinetic spectrometer (Bína et al. 2006) customized for recording of light-induced absorption difference spectra of the bacterial cell suspension in single-shot mode without the necessity of averaging. Activity of RCs was assessed from the light-induced charge separation driven by broadband pulses (~ mJ energy per pulse) of 2 μs duration delivered by a pair of xenon flash lamps positioned at the opposing faces of the sample cuvette, ensuring homogeneous illumination of the sample volume. Absorption spectra were recorded in the 750–980 nm region using a photodiode array detector. The amount of pulse-induced oxidized primary donors (P_870_^+^) was measured by the amplitude of the electrochromic shift of the accessory BChl *a* band at 800 nm (Δ*A*_792_ – Δ*A*_811_). The kinetics of reduction of the primary donor of RC following the single-turnover saturating pulse (Online Resource 1) were collected by repeated measurements with increasing delay between the actinic and the probe pulse. Temperature of the sample during measurement was controlled using thermoregulator TR100 (Photon Systems Instruments Ltd., Brno, Czech Republic).

Difference absorption spectroscopy was also used to obtain temperature of the illuminated sample volume using the amplitude of the absorption band of the second overtone of the –OH stretching at 960 nm (Otal et al. 2003). To avoid overlap of the water signal with the temperature-induced changes of the BChl *a* in the photosynthetic apparatus, we used the absorption difference *A*_954_ − *A*_947_. Linearity of this signal with temperature was confirmed independently by parallel measurement with a thermocouple, yielding a temperature-to-absorbance conversion factor of 5 × 10^−4^ K^−1^. Water absorption was extracted from the spectra collected during the measurement of light-induced absorption changes.

### Respiration measurements

The respiration rate was measured with a Clark-type concentration electrode in a thermostated, magnetically stirred cuvette (Hansatech Instruments Ltd., UK). Dissolved oxygen was analysed at a polarization potential of 700 mV set by OxyCorder-401 (Photon Systems Instruments Ltd., Czech Republic) with a sampling frequency of 30 Hz. Two mL of cell suspension was homogenously illuminated by KL2500 LED cold light source (Schott AG, Germany) providing white light of 200 μmol(photon) m^−2^ s^−1^. The measurement was carried out at 10, 20, 30, 40, 45, 50 and 55 °C. Calibration of the measured signals was carried out against a growth medium equilibrated with the ambient air and then depleted of oxygen by bubbling the chamber with a stream of nitrogen. Tabulated values of oxygen concentration in water at respective atmospheric pressure were used to convert measured signals into oxygen concentration inside the measurement chamber [mmol (O_2_) L^−1^]. The slope of linear regression of a plot of O_2_ conc. versus time normalized to BChl *a* concentration provided the rate of respiration in mmol O_2_ g BChl *a*^−1^ h^−1^.

### Statistical analysis

The least squares numerical fitting was carried out using a custom made MatLab™ script utilizing functions of the Curve Fitting Toolbox (MathWorks Inc., Natick, USA).

## Results

**Fig. 1:**
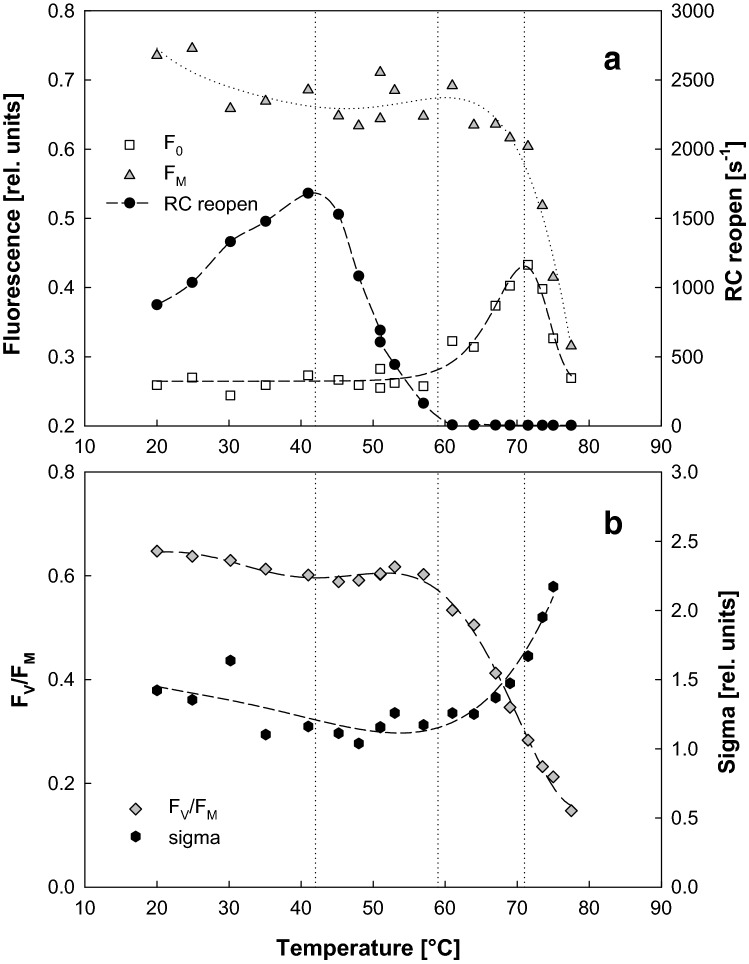
Temperature dependence of fluorescence parameters *F*_0_, *F*_M_ and rate of RC reopening (**a**) and *F*_V_/*F*_M_ and functional cross-section sigma (**b**) recorded in *R. rubrum* cells exposed for 5 min to temperatures between 20 and 78 °C (see “Materials and methods”). The dashed vertical lines represent the different stages of RC inactivation

The activity of photosynthetic RCs was tested in vivo using BChl kinetic fluorometry at temperatures ranging from 20 to 80 °C. Minimum *F*_0_ and maximum *F*_M_ fluorescence, yield of photochemistry (*F*_V_/*F*_M_), and rate of RC reopening were determined for each individual temperature. All the analysed fluorescence parameters displayed large temperature-dependent changes (Fig. 1). The *F*_0_ remained indifferent to the increasing temperature from 20 to 60 °C, but then sharply peaked around 70 °C and rapidly declined (Fig. 1a). *F*_M_ slowly declined between 20 and 42 °C, but then the decline stopped to approx. 65 °C. Beyond this temperature, *F*_M_ rapidly declined reaching the *F*_0_ level at approx. 82 °C. Following changes of minimum and maximum fluorescence, the *F*_V_/*F*_M_ ratio exhibited only a slow minor decline, between 20 and 57 °C, followed by its rapid decrease upon further temperature rise approaching zero at 75 °C. Large variability was observed in the RC-reopening parameter, which steadily rose between 20 and 41 °C (Fig. 1b). At higher temperatures, it started its decline with value of only 10 s^−1^ at 61 °C, asymptotically approaching zero at higher temperatures signalizing the complete inhibition of the electron transport.

**Fig. 2:**
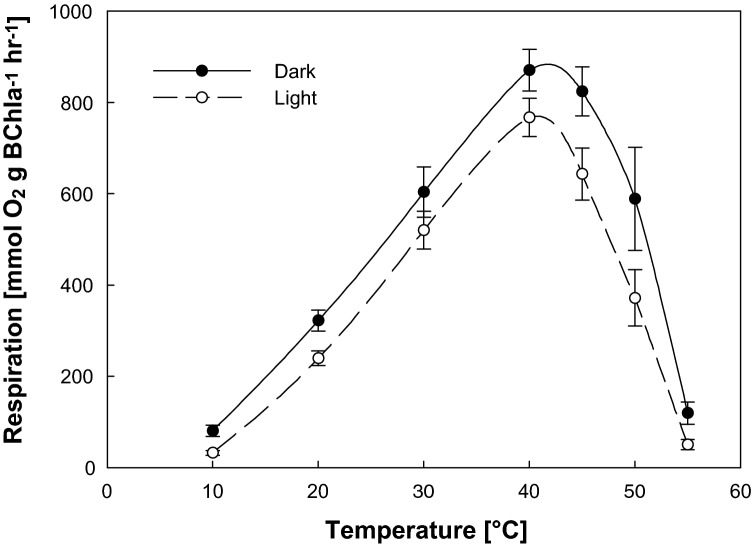
Temperature dependence of respiration of *R. rubrum* grown at 35 °C. The rate of O_2_ respiration was measured in the dark and under illumination of 200 μmol(photon) m^−2^ s^−1^

We wanted to test whether the rate of RC reopening has the same temperature optimum as oxidative phosphorylation. Thus, we determined respiration rate in the dark and also under illumination of 200 μmol(photon) m^−2^ s^−1^ at temperatures from 10 to 55 °C (Fig. 2). The respiration grew linearly between 10 and 40 °C, reaching its maximum around 40 °C. Dark respiration remained high even between the 40 and 45 °C, whereas respiration under illumination was significantly lower at temperatures above 40 °C (*T* test, *n* = 8, *P* > 0.04). At higher temperatures respiration steeply declined, reaching zero activity at 60 °C, which perfectly agreed with the rate of RC reopening determined by BChl fluorometry.

**Fig. 3:**
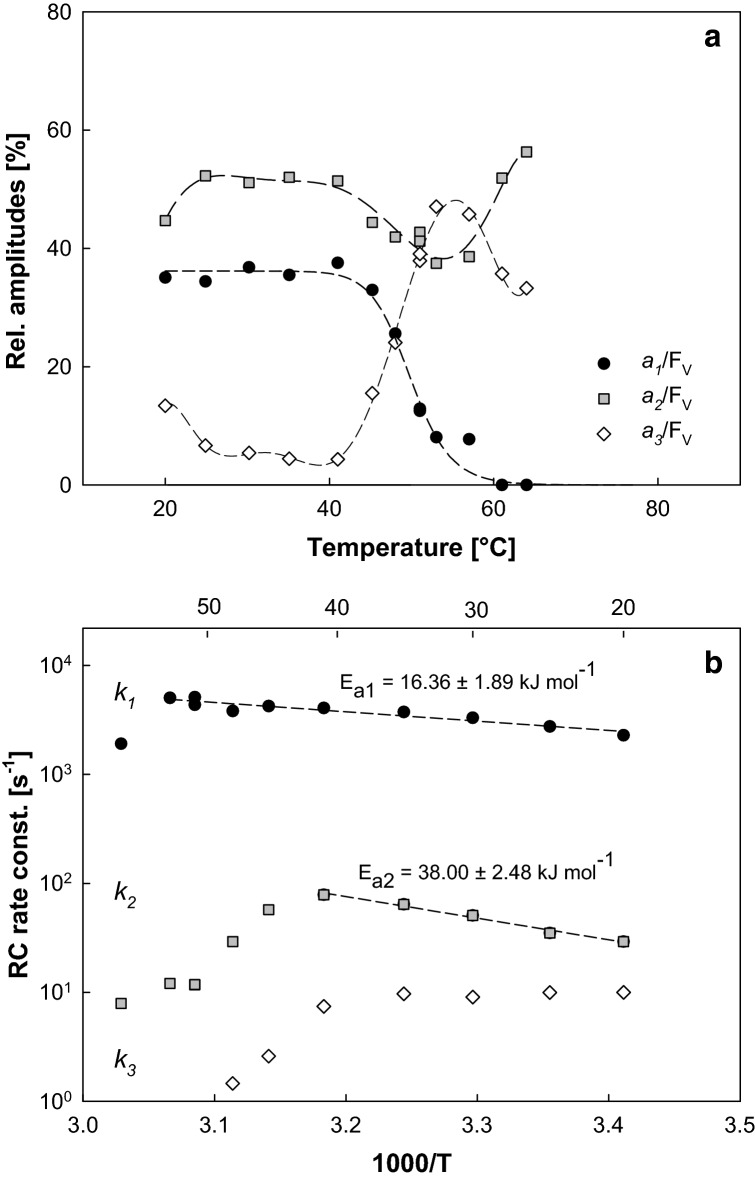
Temperature dependence of fluorescence decay parameters recorded in *R. rubrum* cells exposed for 5 min to temperatures between 20 and 68 °C (see “Materials and methods”). The individual decay kinetics were fitted using three exponentials characterized by amplitude and rate constant. **a** The three amplitudes presented as percentage of variable fluorescence *F*_V_ (the sum of the three amplitudes equals *F*_V_). **b** The three rate constants presented in the Arrhenius plot. The linear fitting of the logarithm of the rate constants was used to calculate activation energies *E*_a1_ and *E*_a2_

To get further information about the electron-transfer activity in the lower (20–41 °C) and middle (41–61 °C) temperature ranges, we looked more into the details of the individual parameters obtained from the fluorescence decay fitting. The kinetics were deconvoluted into three exponential decay kinetics, each characterized by an amplitude *a*_1_, *a*_2_, *a*_3_ and rate constant *k*_1_, *k*_2_, *k*_3_ (see “Materials and methods”). At the investigated lower temperature range the electron transfer was dominated by fast and medium rate kinetics characterized by amplitude *a*_1_ and *a*_2_ (Fig. 3a). The slow kinetics (amplitude *a*_3_) was responsible for approx. 15% of the variable fluorescence at 20 °C, decreasing to its minimum at 41 °C, where it accounted for less than 5% of *F*_V_. The computed rate constants for fast (*k*_1_) and medium kinetics (*k*_2_) rose steadily between 20 and 41 °C, the rapid rate constant *k*_1_ even kept increasing up to 51 °C (Fig. 3b). To analyse the dependence of the rate constants on the temperature, we plotted all the obtained rate constants using the Arrhenius plot which revealed that the fast and medium rate constants follow the Arrhenius equation *k* = *A* exp(− *E*_a_/*RT*), where *A* is pre-exponential factor, *E*_a_ is the activation energy, *T* is absolute temperature in K, and *R* is the universal gas constant. The plots were unimodal across the whole physiological range. From the slope of the logarithmic plots, we determined the activation energies for fast and medium rate constants that were *E*_a1_ = 16.36 ± 1.89 kJ mol^−1^ and *E*_a2_ = 38.00 ± 2.48 kJ mol^−1^, respectively. These values translate to 0.17 ± 0.02 eV and 0.39 ± 0.03 eV for fast and medium rate constants, respectively. The slowest rate component (*k*_3_) was practically activationless exhibiting negative apparent activation energy ranging from − 3 to − 9 kJ mol^−1^.

To aid in the identification of the kinetic components of the BChl fluorescence decay, we performed measurements of the P_870_^+^ decay following a single-turnover flash by transient absorption measurements (Online Resource 1). Measurements with an open and closed sample cuvette incubated in the dark to perform experiments under aerobic and microaerobic conditions, respectively, were done at room temperature. Both in the open and closed systems, P_870_^+^ decayed in two phases: ~ 100 μs and ~ 10 ms. The slower phase was ~ 5 times faster in the closed sample (*k* = 1/(11 ms) vs ~ 1/(50 ms)). Overall, *t*_1/2_ of P_870_^+^ reduction was 2 ms and 20 ms for closed and open samples, respectively. In addition, there was a small (< 15%) contribution of a faster ~ 10 μs phase in the closed sample, which was indistinguishable in the open sample. The comparison of the kinetic parameters obtained from fitting of the BChl fluorescence and P_870_^+^ absorption decays following a single-turnover flash is shown in Table 1.

**Table 1:** Rate constants and respective amplitudes of the individual components of the P_870_^+^ and BChl signal decay following saturating pulse in cells of *Rhodospirillum rubrum* under aerobic conditions

	1/*k*_1_ (μs)	*a*_1_ (%)	1/*k*_2_ (ms)	*a*_2_ (%)	1/*k*_3_ (s)	*a*_3_ (%)
P_870_^+^	380 ± 50	17 ± 2	36 ± 11	76 ± 1	n.d.	n.d.
BChl	396 ± 30.0	37 ± 0.5	31.2 ± 2.43	52 ± 5.6	0.10 ± 0.03	11 ± 5.1

**Fig. 4:**
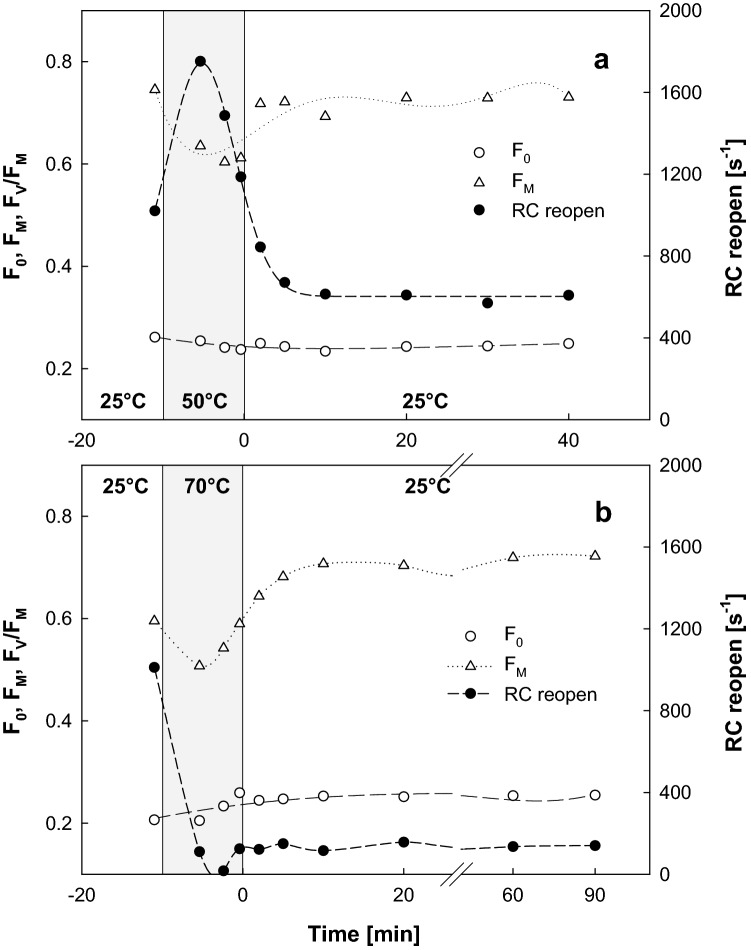
The recovery of fluorescence parameters in the cells exposed to elevated temperature. The cells of *R. rubrum* grown at 25 °C were exposed for 10 min to 50 °C (**a**) or 70 °C (**b**). Then, the cells were brought back to 25 °C, and the recovery of their fluorescence parameters was recorded

The time progress and reversibility of the temperature-induced changes in RC was investigated by measuring flash fluorescence induction–relaxation kinetics of cells exposed to elevated temperatures along two contrasting thermal trajectories. Firstly, cells incubated at 50 °C and 70 °C for an hour were sampled for measurements performed at the incubation temperature every 3 min. The *F*_V_/F_M_ parameter reached a steady level of 0.2 within first minutes of incubation 70 °C and did not change further for up to 60 min. Both *F*_0_ and *F*_M_ values, however, steadily declined beyond the 10 min of incubation at 70 °C until they both reached 57% of their initial value after 60 min. An hour-long exposure to 50 °C had no effect on the *F*_0_ parameter while the *F*_M_ parameter had 90% of its value at room temperature throughout the whole experiment. Secondly, cells were heated only for 10 min and then rapidly cooled down and kept at 25 °C. The progress of the fluorescence parameters measured in cells heated to 50 °C is shown in Fig. 3a. The *F*_0_ signal remained unchanged throughout the experiment whereas the *F*_M_ parameter slightly decreased by 15% as expected (cf. Fig. 1) but fully recovered instantly after cooling down the cells. The rate of RC reopening, however, followed a different trend with only partial recovery during the heating and cooling protocol (Fig. 4a). First, a transient stimulation of 2 min into the 50 °C heating period caused the rate of RC reopening to rise 1.6 times, followed immediately by a gradual inhibition that continued for another 5 min even after the cells were cooled back to 25 °C. The RC-reopening values remained stabilized at 60% of the initial value for another 30 min. Ten minutes of heating of the cells to 70 °C resulted in the increases in both *F*_0_ and *F*_M_ parameters (Fig. 4b). The rate of RC reopening decreased down to zero at the fifth minute of heating and recovered only to 10% of its original value upon cooling back to 25 °C. The rate of RC reopening was slowed down to the intermediate 26% of its initial level upon recovery from the 10 min of heating of cells at 52 °C, whereas both the *F*_0_ and *F*_M_ parameters remained unchanged.

**Fig. 5:**
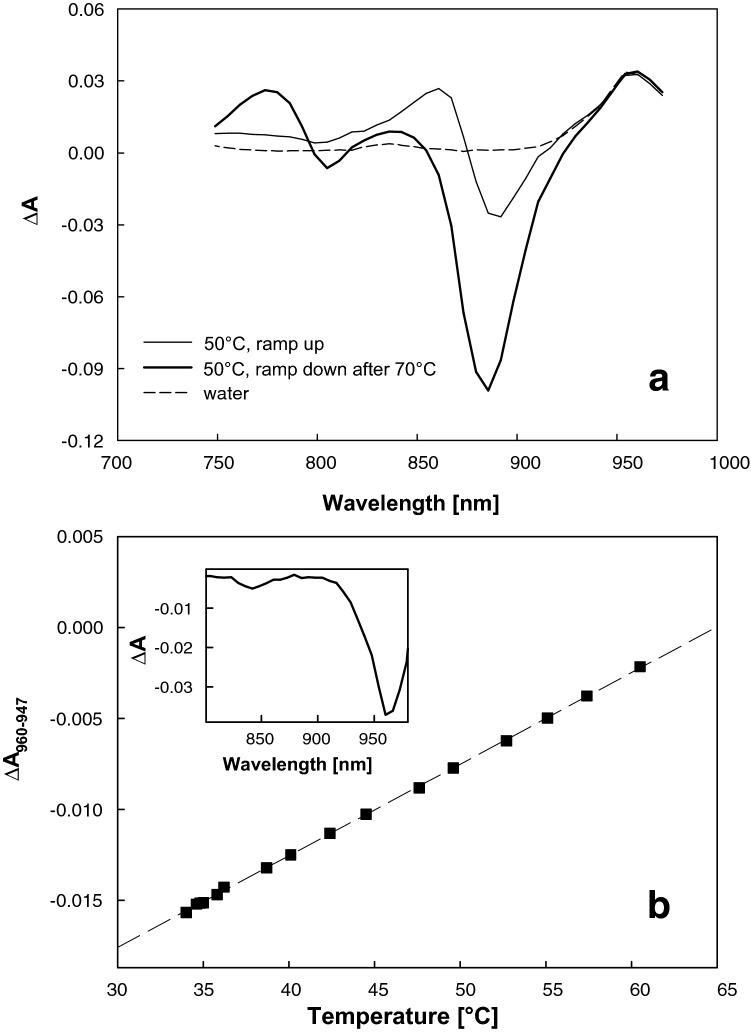
Temperature-induced absorbance changes in whole cells of *R. rubrum*. Spectra correspond to difference between 50 and 23 °C (**a**). First spectrum was recorded during the rising part of the temperature ramp at 50 °C (thin line). It corresponds to fully reversible changes. The second spectrum was taken during the cooling following the 4-min hold at 70 °C (thick line). Difference in absorptions of water corresponding to this temperature step is shown as dashed line. “Water thermometer” (**b**). Data were acquired by simultaneous recording of absorbance spectra and bulk temperature (using a thermocouple immersed in the sample) of a water-filled cuvette. Initial temperature was 65 °C. The cuvette was continuously stirred to ensure homogeneous cooling. Inset: difference in spectra of the water sample after cooling by 30 °C

The gradual decay of the yield of photochemistry, revealed by the ratio of fluorescence parameters *F*_V_/*F*_M_, becomes prominent only in the high temperature range (> 61 °C). The effect of temperature on stability of the primary charge separation in the RC was further studied in detail by difference absorption spectroscopy. In these experiments, the cell suspension was subjected to a temperature ramp, and the activity of RCs was periodically monitored as flash-induced formation of the oxidized primary donor, P_870_^+^. The measurements were taken at 3 µs after the end of the 2 µs-long single-turnover actinic pulse; hence, they corresponded to the stable charge-separated state, P_870_^+^Q_A_^−^. Measurements of the pre-flash absorbance of the samples provided a set of steady-state absorption spectra revealing the temperature-dependent structural integrity of LH1 (Fig. 5). Figure 5a shows the steady-state spectra of the cells’ culture taken at 50 °C during the rising and descending part of the temperature ramp to 70 °C, presented as a difference between the absorbance at the starting temperature, 23 °C, and at 50 °C. The spectra were identical in the region above 940 nm, where the signal was dominated by the water vibration spectrum. The amplitude of this signal was proportional to the temperature (Fig. 5b). Difference absorption spectrum of water is shown for comparison (Fig. 5b, inset). The difference spectrum acquired during the rising part of the ramp features a symmetrical band around 875 nm indicating a hypsochromic shift of the LH1 absorption band. The increasing temperature probably intensifies the disorder of the LH1 leading to weakening of the excitonic interaction responsible for the pronounced red-shift of the BChl *a* spectrum characteristic of the bacterial LH antennae. The effect was reversible, as expected from the symmetry of the difference spectrum that indicated that no loss of the overall dipole moment occurred. In contrast, the 50 °C spectrum taken after the sample was subjected to heating to 70 °C and was dominated by a negative band peaking at ~ 885 nm suggesting dissociation of LH1. This was accompanied by an appearance of a positive band at 780 nm of free BChl *a* demonstrating an irreversible damage. Moreover, a small negative band appeared at ~ 800 nm indicating loss of the accessory BChl in the RC.

**Fig. 6:**
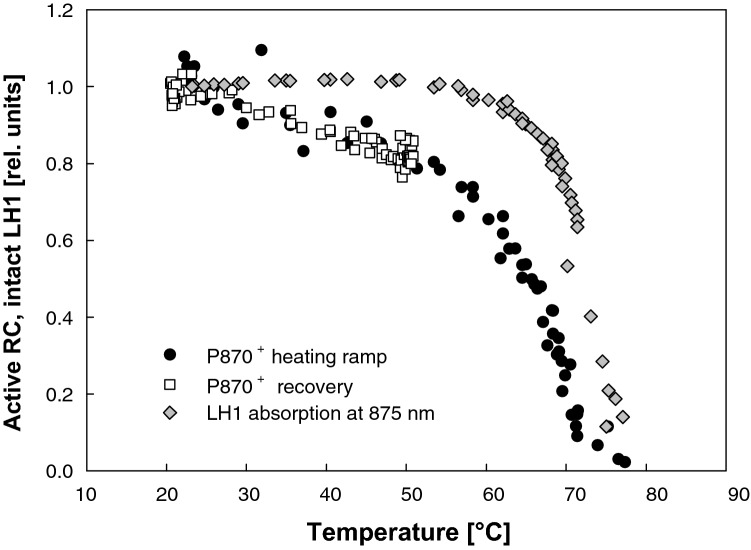
Effect of temperature on primary charge separation and LH1 integrity in whole cells of *R. rubrum*. RC function was measured as yield of single-turnover pulse-induced primary donor (P_870_) oxidation acquired during a temperature ramp from 22 to 75 °C (closed circles). Empty squares represent data acquired in an experiment consisting of ramp from 22 to 50 °C, followed by 10-min hold at 50 °C and then a cooling ramp back to 22 °C. LH1 damage was assessed from bleaching at 875 nm in the temperature-dependent steady-state spectra (grey diamonds). Temperature was measured using the water absorbance at 960 nm

The stabilities of the RC and LH1 were monitored by the temperature dependence of the amplitudes of the absorption changes (Fig. 6) derived from the steady-state difference spectra like those shown in Fig. 5. The RC activity is shown as the amplitude of the absorbance changes pertinent to P_870_^+^ from the flash-photolysis experiment. The extent of LH1 damage was quantified at 875 nm, which corresponded to the isosbestic point of the reversible absorption changes. The value was normalized to the steady-state absorbance of the sample prior to the measurement. As seen in Fig. 6, the activity of the RC slightly decreased upon increasing the sample temperature from 23 to 50 °C, but this decrease was fully reversible even after the sample was held at 50 °C for 10 min. Further increase of the temperature led to a fast loss of RC activity, dropping to 50% at ~ 66 °C, and the charge separation ceased at ~ 75 °C. On the contrary, the LH1 remained stable well above 70 °C with 50% loss occurring only at ~ 72.5 °C.

**Fig. 7:**
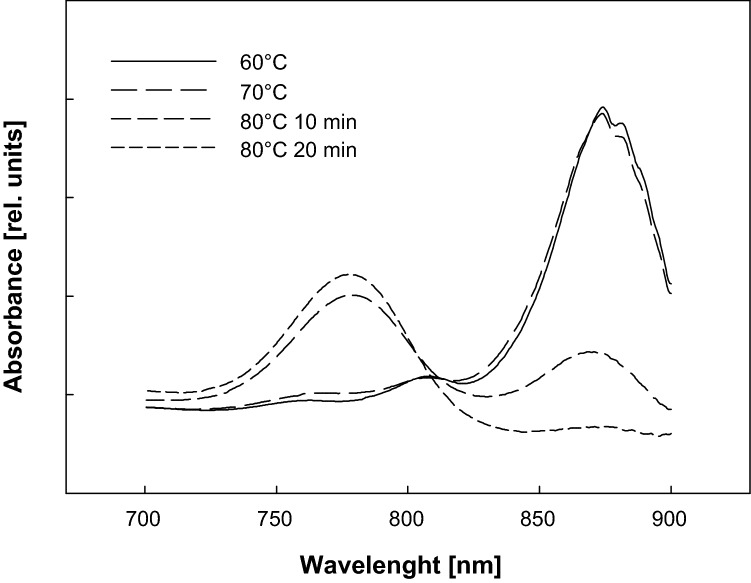
The changes of the NIR absorption spectrums of the *R. rubrum* cells exposed to the elevated temperature for 10 min. At 80 °C the second spectrum was taken after 20 min

The disintegration of the photosynthetic complexes was recorded also using steady spectroscopy (Fig. 7). The near infra-red absorption spectrum of the cells heated to 80 °C shows gradual disappearance of 880 nm absorption band corresponding to light-harvesting antenna complex LH1. Concomitant with the disappearance of 880 nm band there appeared a 786 nm absorption band originating from free BChl *a* molecules (Fig. 7).

## Discussion

In general, temperature has a complex effect on photosynthetic reactions. The primary electron-transfer steps following the light absorption are temperature independent down to 1°K (Arnold and Clayton 1960), whereas the charge recombination of the P^+^Q_A_^−^ state was shown to be activationless in a broad temperature range from 0 to 50 °C (Venturoli et al. 1991). The proton-coupled electron-transfer reactions that follow the charge separation at the acceptor side of the RC are rate limited by protein conformational changes (Kleinfeld et al. 1984; Graige et al. 1998). Increasing the temperature within the physiological range accelerates the rate of the latter reactions exponentially obeying the Arrhenius law. Temperatures past the RC’s optimum increasingly disrupt the structure of the RC (Odahara et al. 2011) and its adjacent LH1 subunits (Helenius et al. 1997) coordinating the cofactors indispensable for the photosynthetic reactions. At temperatures slightly above the optimum, components of the RC undergo structural changes that are still reversible, resulting in a reversibly lowered photosynthetic capacity. Upon a further temperature increase, irreversible changes take place due to protein denaturation, leading to permanent damage of the RC-LH1 complex and finally also the loss of the coordinated pigments.

To disentangle all the effects temperature has on the reaction centre activity, we divided the temperature continuum into four stages and analysed them individually in more detail. The first stage was found between 20 and 41 °C and is characterized by a constant *F*_0_ signal and only slightly decreasing *F*_V_/*F*_M_ ratio, which signalizes the high efficiency of primary charge separation. The rate of RC reopening increased approx. twofold between 20 and 41 °C. The second stage occurred between 41 and 59 °C. It is characterized by a decaying RC reopening parameter, while other fluorescence parameters *F*_0_, *F*_M_, and *F*_V_/*F*_M_ stayed almost constant. The third stage occurred between 60 and 71 °C and was characterized by an increase of *F*_0_ fluorescence and the accompanying decrease of *F*_V_/*F*_M_. The last stage was observed above 71 °C, which was characterized by a rapid decrease of both *F*_M_ and *F*_0_ fluorescence.

The first stage (20–41 °C) represents, clearly, an optimal temperature range with a highly effective primary charge separation. The thermal stability of the primary charge separation characterizes only the rapid photosynthetic processes occurring within the RCs. After stabilization, the generated charges have to be transferred down the electron-transport chain. Figure 3 demonstrates the Arrhenius plots of the components of the BChl fluorescence decay following the saturation light pulse. The two of the fastest components exhibit an activation across the physiological range on temperatures. The first, fastest component actually extends its activation regime to the temperatures above 50 °C. To aid in identification of the these kinetic components, we compared the parameters of BChl fluorescence decay with the P_870_^+^ decay following a single-turnover flash obtained by transient absorption measurements (Table 1 and Online Resource 1). The rate constant *k*_1_ is governed by the kinetics of reduction of P_870_^+^ by the cytochrome c_2_^2+^ (Asztalos et al. 2015) kinetically coinciding with a spectrally silent Q_A_^−^ to Q_B_ first interquinone electron transfer (Okamura et al. 2000). The *k*_2_ rate constant reflects the rate of diffusion limited binding of the Q_B_ to the reaction centre followed by the Q_A_^−^ to Q_B_ first interquinone electron transfer concomitant with the slow reduction of P_870_^+^ by the cyt c_2_^2+^. The rate of recombination of the charge-separated state P_870_^+^Q_A_^−^ is represented by the *k*_3_ rate constant (for details see Online Resource 1).

The rate of RC reopening reached its maximum (i.e. the maximum of the photosynthetic RC activity) at 41 °C (Fig. 1), which corresponds to the maximum of cellular respiration (Fig. 2). It is interesting to note that this maximum is 8 °C above the *R. rubrum* optimum growth temperature which is 33 °C (Kaiser and Oelze 1980a, b). A similar observation was made already before in the case of phototrophic bacterium *Dinoroseobacter shibae* from Rhodobacterales (Kaftan et al. 2019) and mitochondria (Chrétien et al. 2018). It was speculated that the elevated optimum of the electron-transfer activity may be connected with the necessity to cope with the heat dissipated during the photochemical reactions (Kaftan et al. 2019). Our new observation is consistent with this hypothesis.

The second stage (41–59 °C) is mainly characterized by the gradual decay of the RC-reopening rate as well as respiration (Figs. 1, 2). The downfall of the RC-reopening rate (Fig. 1) originates from the disappearance of the amplitude of the rapid component of the BChl decay *a*_1_ (Fig. 3a) that is attributed to the reduction of P_870_^+^ by the cytochrome c_2_^2+^ and electron transfer between the P^+^Q_A_^−^Q_B_ and P^+^Q_A_Q_B_^−^ states. The amplitude of the fastest component of the P_870_^+^ decay is approx. two fold smaller than the one of the BChl fluorescence decay (Table 1). This fact indicates that sizeable fraction of the BChl signals may be governed by the stability of the first interquinone electron transfer at the acceptor side of the RC. Acclimation of the structural stability of the mechanism responsible for gating of the proton-coupled electron transfer at the acceptor side of the RC was proposed to be indispensable for withstanding the elevated temperatures. Such a proposal has been made for all type 2 photosynthetic RC (Shlyk-Kerner et al. 2006) with a partial confirmation of the theory by engineering thermotolerant mutants of cyanobacterium *Synechocystis* PCC6803 (Dinamarca et al. 2011; Shlyk et al. 2017). Here we provide further experimental evidence supporting this claim using in vivo measurements of the temperature dependence of photosynthetic RC activity in *R. rubrum*. The Arrhenius plots of the *k*_2_ and *k*_3_ kinetic components of the BChl decay displayed a linearly decreasing phase within the second temperature stage. The decrease of the reaction rate with the increasing temperature probably results from the thermal perturbation of the protein scaffold increasingly limiting the cofactors, correct distance and respective orientation.

The third stage (59–71 °C) likely represent a gradual inactivation of primary photochemistry within the RCs. This agrees with the observation of the increase of *F*_0_ at about 70 °C (Fig. 1) that can be readily explained by the appearance of photosynthetic complexes, which possess the intact antenna harbouring an inactive RC. This may explain the increase of sigma (Fig. 1b), which likely reflects an effective increase of the absorption cross-section of the remaining active centres by the light harvesting complexes of the neighbour inactive units. The temperature range agrees well with the temperature range 58–67 °C determined for denaturation of L–M subunits in isolated chromatophores of *R. rubrum* (Odahara et al. 2011). For further insight into the thermal deactivation of the charge separation in the RC, we focused on the kinetic absorption measurements of the flash-induced primary donor oxidation. As described in detail in the Online Resource 2, we modelled the time dependence of the temperature-induced loss of charge separation in RC by a Lumry and Eyring (1954) kinetic model of transition from native (*N*) to denatured (*D*) protein via a reversibly formed intermediate (*I*): *N* ⇌ *I* → *D*, where *N* was quantified by the flash-induced P870^+^ and both *I* and *D* were considered inactive in charge separation. Despite being a rather crude approximation of the system, the model successfully captured the behaviour of the system, as shown in Online Resource 2. The fit yielded following values of the (Arrhenius type) activation energies: *E*_a_(*N* → *I*) = 90 ± 13 kJ mol^−1^; *E*_a_(*I* → *N*) = 20 ± 13 kJ mol^−1^; *E*_a_ (*I* → *D*) = 330 ± 30 kJ mol^−1^. In comparison with the activation energies estimated previously for isolated RC (Hughes et al. 2006; Palazzo et al. 2010), the energy of the irreversible step is ~ 1.5–2 times higher. This difference is hardly surprising given the fact that in our case, the native LH1-embedded RC was studied. Also, in the cited work, the RC damage was assessed from the loss of steady-state absorption of the cofactors, not from charge-separating functionality of the electron-transfer chain.

It is noteworthy that the bacterial RC retained stable primary photochemistry even ~ 30 °C above their growth temperature. We observed the same phenomenon also in our recent experiments with phototrophic Rhodobacterales, which also retained fully functional photochemistry up to 60 °C (Kaftan et al. 2019). Similar observations were made also by Watson et al. (2005) in *Rhodobacter sphaeroides*. During their measurements of RC activity, they found that it was almost unaffected up to 50 °C, whereas half of the RCs degraded at 72.1 °C. These authors raised a question as to whether RC of purple bacteria need any special adaptation to support growth at elevated temperatures. Indeed, it seems that bacterial photosynthetic complexes have very good thermostability, probably as a result of the overall robust architecture.

The fourth stage (above 71 °C) represent the decomposition of photosynthetic complexes that occurs at higher temperatures than the inactivation of the RCs (see Fig. 6). This process is manifested by disappearance of both *F*_M_ and *F*_0_ signals (Fig. 1) as well as absorption at 880 nm (Fig. 7). It occurs at temperatures above 71 °C, which corresponds to temperature range 74–86 °C reported for decomposition of LH1 in *R. rubrum* chromatophores (Odahara et al. 2011).

Purple-bacterial antenna complexes are characterized by excitonic coupling of BChl *a* molecules that gives rise to intense absorption bands positioned at energies much lower (corresponding to ~ 100 nm in case of LH1) than the absorption of free BChl *a* in solution. This coupling is extremely sensitive to mutual orientation of transition dipoles of the interacting molecules. Our data shows that the LH1 of mesophilic organisms is capable of maintaining the spatial organization of pigments at temperatures that exceed the growth temperature by 40 °C. The slight blue shift of the LH1 absorption observed at sub-lethally increased temperatures can be readily explained by increased structural disorder affecting the pigment orientation. A similar observation was made on the LH2 antenna by Rätsep et al. (2018). It is likely that this disorder also affects the coupling of the antenna pigments to the RC, leading to a slight (reversible) decrease of yield of photochemistry at temperatures below 60 °C. Present results document that the ability of the LH1 antenna to support the excitonic structure is lost only upon degradation of the complex with concomitant release of the pigment, hence this sturdiness is the universal feature of the purple-bacterial antenna rings.

In conclusion, we report that the photosynthetic apparatus of Alphaproteobacterium *R. rubrum* exhibits good thermostability. Their electron transport as well as respiration operates at a maximum rate at 41 °C, at higher temperatures both activities decline. Interestingly, the primary charge separation and forward electron stays functional up 60 °C, approx. 30 °C above the optimum growth temperature. Finally, the photosynthetic complexes physically decompose at temperatures above 70 °C. The extended temperature optimum of photosynthetic reaction centre in a representative species of Rhodospirillales matches and complements the activities observed in Rhodobacterales and Sphingomonadales reported earlier (Kaftan et al. 2019). Here we provide the final evidence the high thermostability of the photosynthetic reaction centres is present across all Alphaproteobacteria.

## Electronic supplementary material

Below is the link to the electronic supplementary material.

Supplementary material 1 (PDF 344 kb)

Supplementary material 2 (PDF 786 kb)
