# Supplementary material for: Temperature dependence of photosynthetic reaction centre activity in Rhodospirillum rubrum
Source: Photosynth Res. 2019 Jul 2;142(2):181–93. doi: 10.1007/s11120-019-00652-7 (PMC6848049; doi:10.1007/s11120-019-00652-7)
Supplement: Supplementary file 1 — Supplementary material 1 (PDF 344 kb) [file 11120_2019_652_MOESM1_ESM.pdf]

Online Resource 1

Temperature dependence of photosynthetic reaction centre activity in *Rhodospirillum rubrum*

David Kaftan[a,b,*], David Bína[b,c], Michal Koblížek[a,b]

[a]*Center Algatech, Institute of Microbiology CAS, CZ-37981 Třeboň, Czech Republic*

[b]*Faculty of Science, University of South Bohemia, CZ-37005 České Budějovice, Czech Republic*

[c]*Biology Centre, Czech Academy of Sciences, Branišovská 31, České Budějovice, Czech Republic*

[*]Corresponding author: david.kaftan@prf.jcu.cz; phone: +420 387776230

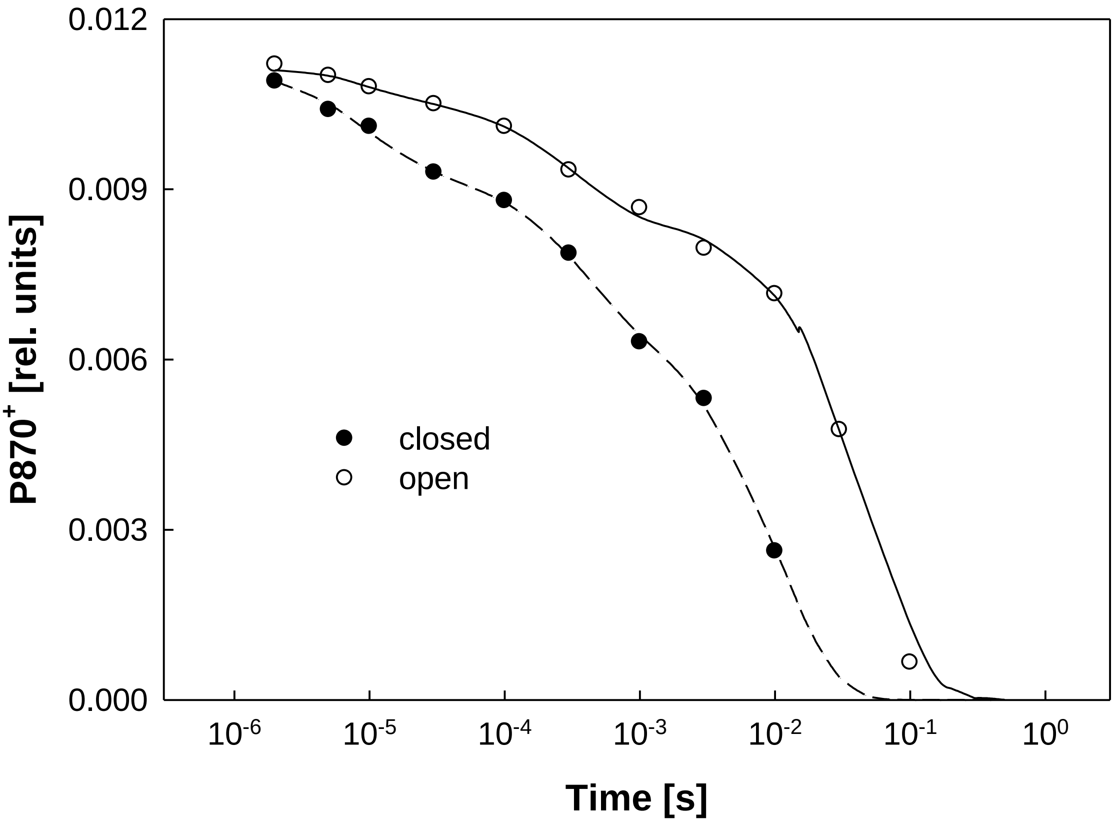

Supplementary Figure 1 Decays of oxidized primary donor, P_{870}^+, following a 2μs single-turnover Xe pulse were measured in suspensions of whole cells of purple bacteria under aerobic ('open') and microaerobic ('closed') conditions. Microaerobic conditions were achieved by pre-incubating the cells in the dark for more than 30 minutes in closed cuvettes.

Supplementary discussion of the fluorescence decay components identity

The first exponential decay component is characterized by rate constant k_1 and amplitude a_1. The rate of first interquinone electron transfer reaction reported elsewhere (Graige et al. 1998, Okamura et al. 2000) that are based on the absorption changes (ΔA_{412}, ΔA_{757}) are reasonably close to the determined values of the k_1. The transition from $P^+Q_A^-Q_B$ to $P^+Q_AQ_B^-$ state has however only minor effect on BChl fluorescence yield. The generally accepted interpretation of the P_{870}^+ absorption changes at the time scale of ~100μs (that correspond to the k_1) is the

reduction of P_{870}^+ by the cyt c_2 (Asztalos et al. 2015). This process is always associated with small activation energies bellow 10 kJ mol^{-1} in *Rhodobacter sphaeroides* (Venturoli et al. 1993) and *Rubrivivax gelatinosus* that have cyt c_2 closely associated with their RC. In our case, the rate constant derived from decay of BChl fluorescence exhibits an acceleration with increasing temperature and an activation energy of 16 kJ mol^{-1}. Slightly larger activation energy may reflect the binding of the loosely associated cyt_2 with the reaction centre of *Rhodospirillum rubrum*. In conclusion, the k_1 rate of BChl fluorescence decay corresponds to the fast reduction of oxidized primary donor P_{870}^+ by the cyt c_2^{2+} along with a spectrally silent Q_A^- to Q_B first interquinone electron transfer.

The second rate component of the BChl fluorescence decay k_2 (time constant of 30-100 ms) kinetically overlaps with the slow component of the decay of the P_{870}^+ signal (20-40 ms). A comparison with the literature suggests that k_2 is mostly determined by quinone diffusion in the membrane. Further support for this can be found in the oxygen dependence of this rate, as it can be expected that anaerobic conditions will lead to reduction of the membrane Q_B pool. Finally, it is also supported by the observed temperature dependence: the rate constant derived from decay of BChl *a* fluorescence exhibits significant acceleration with increasing temperature and large activation enthalpy of 35 kJ mol^{-1}. This is comparable to reported activation energies of up to 50 kJ mol^{-1} (Chazotte and Hackenbrock 1989) of ubiqionone diffusion in mitochondrial membrane, a limiting step for the mitochondrial electron transport. It is also important to note, that the RC of *Rhodospirillum rubrum* is only loosely associated with its cyt c_2 (Paddock et al. 2005). The k_2 rate constant therefore reflects the rate of diffusion limited binding of the Q_B to the reaction centre followed by the Q_A^- to Q_B first interquinone electron transfer concomitant with the slow reduction of P_{870}^+ by the cyt c_2^{2+}.

The slowest rate component of the bacteriochlorophyll fluorescence decay k_3 (time constant of 1s) is not traceable in our measurements of the P_{870}^+ signal. This slow process

accounts to less than 10% of the amplitude of the BChl fluorescence decay within the physiological range of temperatures. While exhibiting small and negative activation enthalpy of -3 to -9 kJ mol^{-1}, its amplitude suddenly rises at temperatures above 40°C in a reciprocal manner to the fastest component of the BChl *a* fluorescence decay k_1 and also the rate of RC reopening. Therefore, we interpret the k_3 reaction rate constant as referring to the rate of recombination of the charge separated state P_{870}^{+} Q_A^{-}.

References

Asztalos E, Sipka G, Maróti P (2015) Fluorescence relaxation in intact cells of photosynthetic bacteria: donor and acceptor side limitations of reopening of the reaction center. Photosynth Res 124: 31–44

Chazotte B, Hackenbrock CR (1989) Lateral diffusion as a rate-limiting step in ubiquinone-mediated mitochondrial electron transport. J Biol Chem 264(9): 4978–4985

Graige MS, Feher G, Okamura MY (1998) Conformational gating of the electron transfer reaction Q_A^{-} Q_B –> $Q_AQ_B^{-}$ in bacterial reaction centers of *Rhodobacter sphaeroides* determined by a driving force assay. Proc Natl Acad Sci USA 95: 11679–11684

Okamura MY, Paddock ML, Graige MS, Feher G (2000) Proton and electron transfer in bacterial reaction centers. Biochim Biophys Acta 1458: 148–163

Paddock ML, Weber KH, Chang C, Okamura MY (1995) Interactions between cytochrome c_2 and the photosynthetic reaction center from *Rhodobacter sphaeroides*: the cation–π interaction. Biochemistry 44: 9619–9625

Venturoli G, Trotta M, Feick R, Melandri BA, Zannoni D (1991) Temperature dependence of charge recombination from the $P^+Q_A^-$ and $P^+Q_B^-$ states in photosynthetic reaction centers

isolated from the thermophilic bacterium *Chloroflexus aurantiacus*. Eur J Biochem 202: 625–634.
