# Supplementary material for: Temperature dependence of photosynthetic reaction centre activity in Rhodospirillum rubrum
Source: Photosynth Res. 2019 Jul 2;142(2):181–93. doi: 10.1007/s11120-019-00652-7 (PMC6848049; doi:10.1007/s11120-019-00652-7)
Supplement: Supplementary file 2 — Supplementary material 2 (PDF 786 kb) [file 11120_2019_652_MOESM2_ESM.pdf]

Online Resource 2

Temperature dependence of photosynthetic reaction centre activity in *Rhodospirillum rubrum*

David Kaftan[a,b,*], David Bína[b,c], Michal Koblížek[a,b]

[a]*Center Algatech, Institute of Microbiology CAS, CZ-37981 Třeboň, Czech Republic*

[b]*Faculty of Science, University of South Bohemia, CZ-37005 České Budějovice, Czech Republic*

[c]*Biology Centre, Czech Academy of Sciences, Branišovská 31, České Budějovice, Czech Republic*

[*]Corresponding author: david.kaftan@prf.jcu.cz; phone: +420 387776230

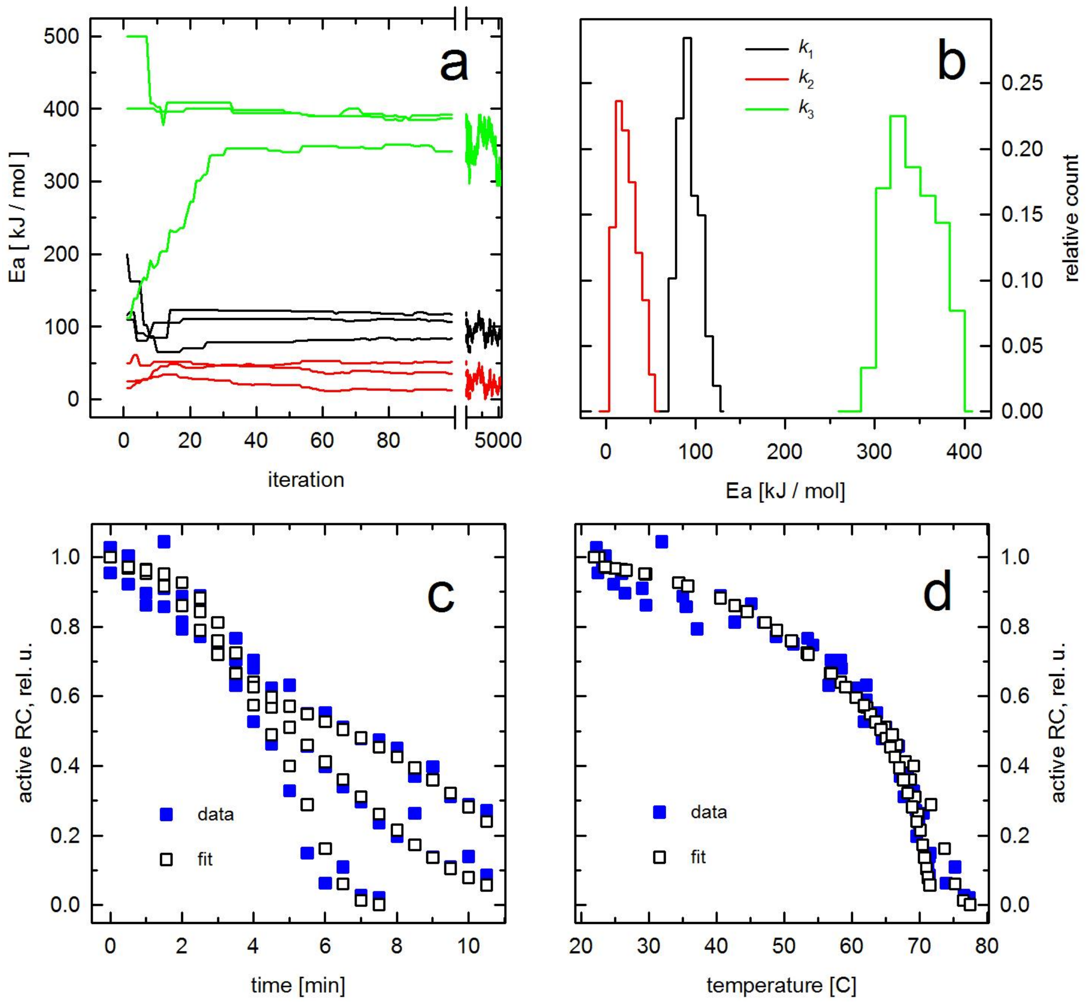

Supplementary Figure 2 Fits of the RC deactivation data by means of the following kinetic scheme: $N \rightleftharpoons I \rightarrow D$, where N corresponds to active, charge-separating RC by means of the MCMC algorithm; **a** – examples of three optimization trajectories; **b** – resulting distributions of the activation energies corresponding to the rate constants given in the legend, these corresponds to k_1: $N \longrightarrow I$, k_2: $I \longrightarrow N$, k_3: $I \longrightarrow D$; **c** – time-dependence of the relative concentration of active RC as a function of time. Data (blue symbols) come from three independent measurements. Empty symbols show the fit results; **d –** a different presentation of the data in **c,** showing the temperature dependence of the active RC, along with the fit results. These data correspond to the Fig. 6 in the main text.

Supplementary analysis of the thermal inactivation of the native RC-LH1

In this section we describe the analysis of the thermal inactivation of the native RC-LH1 complex as monitored by the absorption measurements of stable flash-induced charge separation. The analysis is based on the basic Lumry-Eyring model (Lumry and Eyring 1954) for protein denaturation:

$$N \rightleftharpoons I \rightarrow D, \qquad \text{A1}$$

where the transition from the native (*N*) to final, denatured (*D*), state involves a reversibly formed intermediate (*I*). While such scheme is clearly a major simplification of the native system consisting of a membrane-embedded multiprotein complex, it offers a tractable description of fundamental properties of our sample and was previously successfully applied to analysis of liposome-embedded RC (Hughes et al. 2006). The kinetic scheme can be represented by a simple system of differential equations, where *N* is for simplicity equated with the concentration of active, charge-separating RC:

$$\begin{aligned} \dot{N} &= -k_1 N + k_2 I \\ \dot{I} &= k_1 N - (k_2 + k_3) I. \end{aligned} \qquad \text{A2}$$

Kinetics of the inactivation is determined by the three rate constants which can be assumed to follow an Arrhenius type dependence on temperature, which in the present case is also time-dependent, T [K] = $T(t)$:

$$k_i(t) = A_i \exp\left(-\frac{E_a}{RT(t)}\right), \qquad \text{A3}$$

resulting in the time-dependence of the rate constants $k_{i=1,2,3}$ in the model A2. Given the instrumental limitations of our setup, the analysis cannot rely on the assumption of constant heating rate, invoked in scanning calorimetry studies. However, since the shape of temperature ramp $T(t)$ within the illuminated sample volume is known from the measurement, it can be entered into the expression for the rate constants, A3, and the kinetics of the RC inactivation can be obtained by full numerical integration of the model A2 without any assumption regarding the equilibrium behavior of the system, although we do assume a fast mixing of the sample at any set $T(t)$. Then, the computed kinetics of N can be fitted to the measured relative concentration of active RC to obtain the activation energies (E_a) of the individual steps characterized by the rate constants $k_{1\text{-}3}$.

Altogether, the model contains six unknowns (3 rate constants and 3 preexponential factors) which we determined using the Markov Chain Monte Carlo approach (Robert and Cassela 2010). Briefly, a random walk over parameter space was performed where the values of the fitted parameters in each step were drawn from symmetric normal distributions (Metropolis sampler). The quality of fit at each iteration was evaluated by sum of squared errors (χ^2). Each proposed move was accepted if $\exp(\chi^2_{(j)} - \chi^2_{(j+1)}) > p$, where p is a random number drawn from <0,1>, i.e. steps toward a better fit, $\chi^2_{(j)} > \chi^2_{(j+1)}$, were always accepted, steps in opposite direction were the less likely accepted the more they decreased the fit quality. At each accepted step, the means of the proposal distribution were changed to the new parameter values. Independence of the result on the initial selection of fit parameters was evaluated by comparing several simulation runs starting from different initial parameters values. To illustrate the algorithm performance, several sample runs are shown in the Supplementary Figure 2. The

analysis was performed using locally written Matlab® (MathWorks, USA) scripts. The resulting posterior distributions for activation energies are shown in Supplementary Figure 2, pane b and the data fits in panes c and d. First 250 iterations were excluded from the final datasets (burn-in). For the purpose of this analysis, data from three independent analyses, representing three slightly different temperature ramps were combined and fitted together.

References

Hughes AV, Rees P, Heathcote P, Jones MR (2006) Kinetic analysis of the thermal stability of the photosynthetic reaction center from *Rhodobacter sphaeroides*. Biophys J 90:4155-4166

Lumry R, Eyring H (1954) Conformation changes of proteins. J Phys Chem 58:110-120

Robert CP, Casella G (2010) Introducing Monte Carlo Methods with R. Springer-Verlag New York
